# A Multilevel Analysis of Perceived Noise Pollution, Geographic Contexts and Mental Health in Beijing

**DOI:** 10.3390/ijerph15071479

**Published:** 2018-07-13

**Authors:** Jing Ma, Chunjiang Li, Mei-Po Kwan, Yanwei Chai

**Affiliations:** 1 Beijing Key Laboratory for Remote Sensing of Environment and Digital Cities, Faculty of Geographical Science, Beijing Normal University, Beijing 100875, China; jing.ma@bnu.edu.cn; 2 College of Urban and Environmental Sciences, Peking University, Beijing 100871, China; lcjiang@pku.edu.cn; 3 Department of Geography and Geographic Information Science, University of Illinois at Urbana-Champaign, Natural History Building, MC-150, 1301 W Green Street, Urbana, IL 61801, USA; mpk654@gmail.com; 4 Department of Human Geography and Spatial Planning, Faculty of Geosciences, Utrecht University, P.O. Box 80125, 3508 TC Utrecht, The Netherlands

**Keywords:** noise pollution, mental disorders, built environment, multilevel model, China

## Abstract

With rapid urbanization and increase in car ownership, ambient noise pollution resulting from diversified sources (e.g., road traffic, railway, commercial services) has become a severe environmental problem in the populated areas in China. However, research on the spatial variation of noise pollution and its potential effects on urban residents’ mental health has to date been quite scarce in developing countries like China. Using a health survey conducted in Beijing in 2017, we for the first time investigated the spatial distributions of multiple noise pollution perceived by residents in Beijing, including road traffic noise, railway (or subway) noise, commercial noise, and housing renovation (or construction) noise. Our results indicate that there is geographic variability in noise pollution at the neighborhood scale, and road traffic and housing renovation/construction are the principal sources of noise pollution in Beijing. We then employed Bayesian multilevel logistic models to examine the associations between diversified noise pollution and urban residents’ mental health symptoms, including anxiety, stress, fatigue, headache, and sleep disturbance, while controlling for a wide range of confounding factors such as socio-demographics, objective built environment characteristics, social environment and geographic context. The results show that perceived higher noise-pollution exposure is significantly associated with worse mental health, while physical environment variables seem to contribute little to variations in self-reported mental disorders, except for proximity to the main road. Social factors or socio-demographic attributes, such as age and income, are significant covariates of urban residents’ mental health, while the social environment (i.e., community attachment) and housing satisfaction are significantly correlated with anxiety and stress. This study provides empirical evidence on the noise-health relationships in the Chinese context and sheds light on the policy implications for environmental pollution mitigation and healthy city development in China.

## 1. Introduction

Noise pollution in cities is an important environmental risk that is detrimental to people’s health and well-being [1]. The World Health Organization (WHO) reported in 2011 that about 1.0–1.6 million disability-adjusted life-years (DALYs) were lost every year in Europe due to noise pollution [2]. More than 30% of the population in Europe was exposed to road traffic noise louder than 55 dB during the night, which would cause severe sleep disturbance and adverse health effects [3]. In China, noise pollution resulting from the rapid urbanization, industrialization and increase in car ownership has become a serious environmental problem, and there is increasing concern over it among the general public and policymakers.

In China, some prior studies found that the L_eq24h_ values (Leq—equivalent 24-h noise level) were higher than 70 dB in Beijing and the majority of urban residents were influenced by excessive noise [4]. Construction noise has become the second most serious acoustic pollution in many Chinese cities, which could cause significant DALYs loss, health damage and social costs [5]. In particular, the road traffic noise in Beijing far exceeded the standard value of noise level and spread outwards with urban sprawls as well as the construction of ring roads [6,7]. The 2016 Reports on the State of the Environment in China showed that approximately 50% of functional zones where highways, railways or urban roads crossing were exposed to high noise pollution during night-time [8]. It suggested that community noise and nocturnal traffic noise were important environmental risk factors to public health. However, research on the noise-health relationship in a rapidly urbanizing context like China has been quite scarce to date [9,10,11].

Much literature on noise pollution exists predominantly in developed countries, particularly in Europe. It provides empirical evidence on the adverse effects of noise pollution on public health in residential areas [12]. Noise has been identified as one of the main environmental stressors that have adverse psychological and physiological effects on human health, including annoyance, cardiovascular and metabolic diseases, sleep disturbance, hearing loss and tinnitus, birth outcomes, and cognitive impairments [13,14,15,16,17,18,19]. However, compared to the physical health or physiological effects which have received considerable attention in academia, the relationship between noise-pollution exposure and population’s mental health or mental disorders has only received modest attention, and the results are inconclusive [20,21,22,23]. 

Mental disorders are a major health risk for urban populations [24]. It was estimated that one in every four persons worldwide would be affected by mental disorders during their lifetime [25]. In China, mental and behavioral disorders were responsible for a large proportion of all years lived with disability (YLDs) in 2010, while major depression was the second leading cause of YLDs [26]. Regarded as a part of the indirect pathway from noise to health, examining the noise effects on mental health or psychological disorders can also be helpful for a better understanding of the influences of noise on physical health, e.g., cardiovascular disease [27].

Aircraft noise, the focus in early research, was found to cause some mental disorders among different populations, such as annoyance, depression, anxiety, nervousness, as well as sleep disturbance [28,29,30]. Recent studies have attempted to extend the noise sources to include traffic noise from roads and railways as well [31,32,33,34,35]. In particular, much research has investigated road traffic noise in different contexts and its correlation with population’s health and wellbeing [36,37]. Studies have shown that higher exposure to road traffic noise could significantly increase annoyance and have harmful effects on people’s sleep quality, wellbeing and other health outcomes [38,39,40]. 

Objective measures of noise pollution have been used in some studies to quantify the health effects of traffic noise in different contexts using various statistical methods [13,14,15]. Prior comparative studies showed that annoyance was associated with objectively measured noise level in noisy streets, but not in quiet streets [41,42]. Meta-analyses of noise and health suggested that night-time road traffic noise was positively associated with sleep disturbance, as well as aircraft noise and railway noise [15,43]. Recent years have also seen a growing number of empirical studies on the health effects of subjectively reported community or neighborhood noise [13,14,15,16,17,18]. Perceived neighborhood noise was found to be associated with poor self-rated health and some mental health symptoms (e.g., depression) in Delhi, India [44,45].

However, although some studies provide relatively consistent evidence on the effects of noise on more serious health outcomes (e.g., ischemic heart disease, hypertension), the evidence concerning various mental health symptoms (e.g., depression) or general health outcomes has been inconclusive [46,47,48]. While some studies have shown a significant relationship between annoyance from a few noise sources and anxiety and depression [28,49], other cohort research reported weak associations between traffic noise exposure and mental health assessed by SF-36 [50] and no significant association or linear trend for psychiatric disorders [51]. Concerning children’s mental health, the results have also been equivocal. Traffic noise exposure and increased noise annoyance were linked to deteriorations of children’s mental health as well as behavioral and emotional symptoms in some cross-sectional analyses [52,53,54], while a longitudinal study reported no significant associations between aircraft noise level and psychological health [55]. 

In addition to objective or subjective measures of noise-pollution exposure, characteristics of the physical built environment, such as housing quality, proximity to green space, land-use mix, and accessibility to public transit, have also been found to be covariates of mental health symptoms or mental disorders, such as stress and depression [56,57,58,59]. Moreover, social environment like social contact or community attachment can influence population’s mental health as well [60,61]. Nonetheless, such variables of the physical built environment and social environment have rarely been taken into account simultaneously in prior research on noise pollution and mental health, particularly in developing countries like China [62].

To conclude, the results on the associations between mental disorders and noise pollution from multiple sources (e.g., road traffic, railways, industries, neighbors, and aircraft) have been inconsistent to date [21,59,63,64]. This is possibly because these studies used different concepts of mental health and different measures (e.g., objective versus subjective) of noise-pollution exposure, and most of them are ecological or cross-sectional studies with poor adjustments in the statistical models and no mediator/moderator variables in the analysis. Relatively few empirical attempts have been made so far to consider multiple noise pollution sources simultaneously and explore their relationships with a wide range of mental health outcomes, while controlling for various confounding factors such as socio-demographics, physical environment, and social environment. 

**Figure 1:**
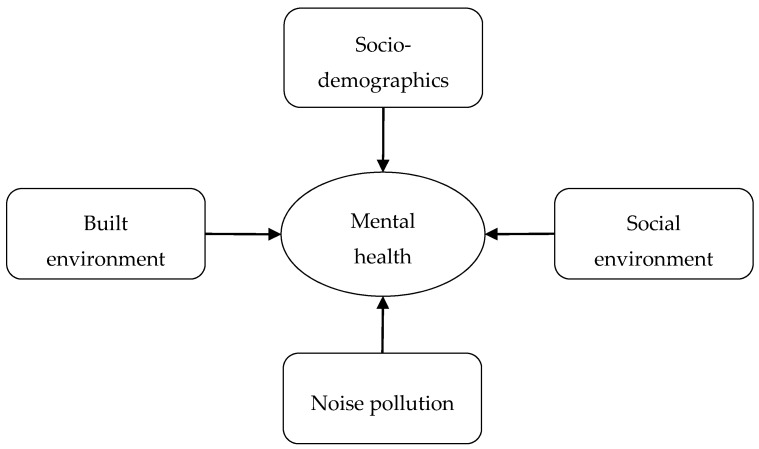
Conceptual framework.

In this study, we aim to extend the literature by exploring the socio-spatial variations of diversified noise pollution in Beijing and developing a comprehensive conceptual framework to examine the relationships between noise pollution and self-reported symptoms of mental health, taking into account a wide range of objective and subjective measures [47,65]. As shown in Figure 1, we consider the mental health correlations with socio-demographics, social environment, objective measures of the physical built environment, and perceived pollution exposure to multiple noise sources, including road traffic, railways (or subways), commercial services (e.g., restaurants, shops), and housing renovation and construction work, which have rarely been examined together in the Chinese context. Moreover, we measure a wide range of self-reported symptoms of mental health in the analysis, including anxiety, stress, fatigue, headache, and sleep disturbance, to understand the connections between noise pollution and urban residents’ mental disorders in Beijing, China.

Drawing upon a health survey conducted in 26 communities of Beijing in 2017, we for the first time demonstrated the spatial distributions of noise pollution from multiple sources at the community level in Beijing. Then we employed rigorous Bayesian multilevel logistic models to investigate the associations between self-reported mental disorders and a wide range of objective and subjective measures, including socio-demographics, social environment, objective physical built environment, perceived noise-pollution exposure, as well as geographic context (or unobserved community effect). We aim to present an empirical noise-health study using rigorous statistical analysis based on a comprehensive conceptual framework and make a timely contribution to a better understanding of the mental health–noise relationships in urban China.

## 2. Data and Variables

### 2.1. Data Sources

Beijing has experienced rapid urbanization, industrialization and increase in car ownership in recent decades, subjecting people to a wide range of environmental hazards and health risks [66]. In this study, we adopted a health survey conducted in Beijing in 2017 to investigate residents’ evaluation of various types of noise pollution in their surrounding environment and their associations with self-reported symptoms of mental health. The survey was implemented by research teams at Peking University and Tsinghua University from March to May in 2017, using a spatial stratified random sampling strategy. First, 26 communities were selected based on their spatial location, housing condition, community type and built environment. These selected communities are representative of the diversified urban neighborhoods in Beijing, including work-unit compounds, commodity housing neighborhoods, affordable housing and mixed neighborhoods. 

Then, in each neighborhood, approximately 50 household members aged between 18 and 65 years old were randomly selected to answer the questionnaire, which contains much information on the household and individual socio-demographics, social networks, evaluations on housing and community environment, noise pollution, and physical and mental health status. In total, surveys of 1280 individuals from 26 communities were collected with valid answers. Here, our analysis comprised 1125 respondents with valid and complete information on socio-demographics, housing conditions and evaluations on noise pollution and mental health. Table 1 presents the distribution of key socio-demographic attributes and variables on housing satisfaction and community attachment in the survey. The majority of participants were married, employed, local residents, house owners, and satisfied with their housing conditions. About 75% of the residents rated the traffic congestion near the community as serious or very serious, while about 63% reported they had feelings of community attachment or belongings. 

In addition to the health survey, point of interest (POI) data in Beijing and GIS-based spatial analysis have also been employed to derive multi-dimensional measures of objective built environment characteristics for each surveyed neighborhood (Table 2). Four aspects of the built environment are considered at the neighborhood scale: public transit accessibility (measured by the distance from each surveyed neighborhood to the nearest subway station), road connection (calculated by the distance to the nearest main road), accessibility to facilities (measured by the distance to the nearest restaurant) and proximity to park. As shown in Table 2, the surveyed communities are characterized by different configurations of these four characteristics. Moreover, these variables are also considered as proxies of objective measures of pollution exposure to various noise sources, such as railways, road traffic, and commercial services. As these objective measures of built environment characteristics might have influences on residents’ mental health, they are included in the statistical analysis and transformed to a standard Normal distribution to reduce the potential problem of heteroscedasticity. 

### 2.2. Measuring Noise Pollution

While the objective data on noise pollution is usually not available at the fine spatial resolution in Beijing in particular and in China more generally, we derived individuals’ subjective evaluations of multiple noise sources in their surrounding environment from the surveys. Perceived noise pollution considered in this study focuses on four dimensions: road traffic noise, railway or subway noise, commercial noise (from restaurants, shops, and other commercial establishments) and housing renovation/construction noise. Therefore, perceived pollution exposures to various types of noise are assessed by the following questions: How would you evaluate the levels of noise pollution (from road traffic, railways or subways, commercial facilities, and housing renovation or construction, respectively) in your neighborhood? Answers are given on a 5-point Likert scale ranging from 1 (very low noise level) to 5 (very high noise level).

**Figure 2:**
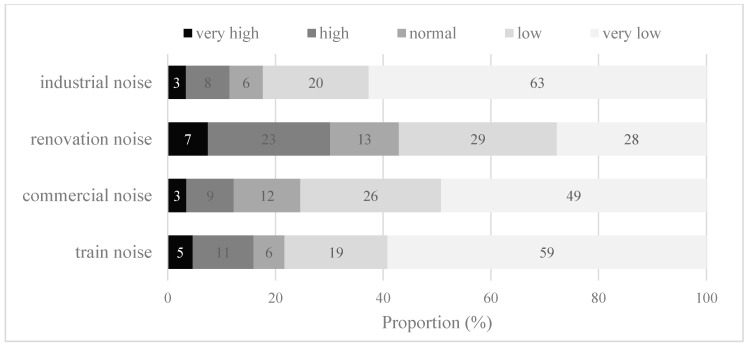
Population (%) in perceived noise-pollution levels of different categories.

Figure 2 presents the proportions of each category in various types of noise-pollution exposures perceived by survey respondents. A noticeable variation exists between the proportions of each category for the four types of noise pollution. The percentages of urban residents rating the levels of housing renovation noise pollution and road traffic noise pollution in their neighborhoods as high or very high are 30% and 37% respectively. In contrast, there are only 12% and 16% of residents reporting serious commercial and railway noise pollution in their surrounding environment. This suggests that road traffic and housing renovation are the principal sources of noise pollution in Beijing, possibly due to high usage of private cars and the prosperous second-hand housing market in China’s capital city.

**Figure 3:**
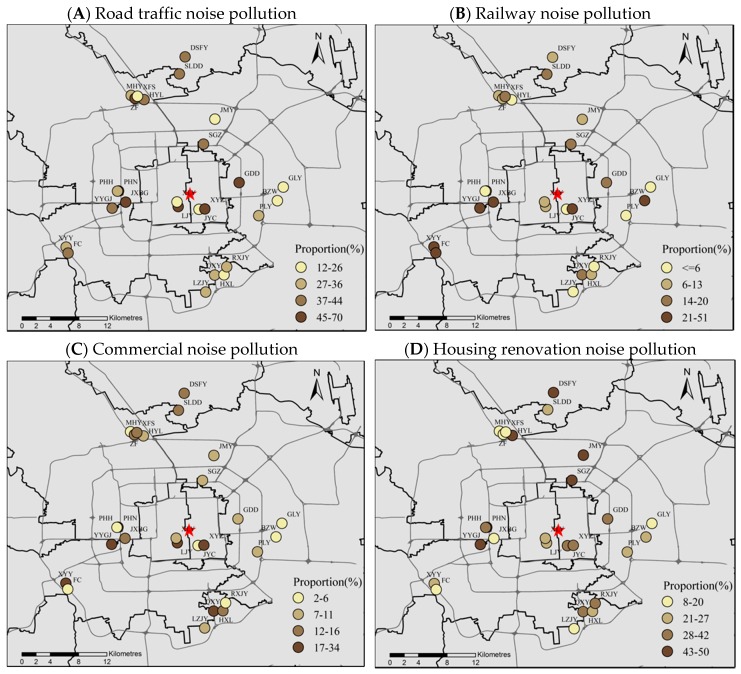
Spatial distribution of the population (%) reporting high or very high noise pollution at the community level in Beijing.

Moreover, we further recoded the original noise-pollution variables into three categories: 1 for no or very low noise pollution, 2 for moderate noise pollution (including low and normal), and 3 for high noise pollution (including high and very high). Figure 3 shows, by quantile, the spatial distributions of the respondents (%) reporting high pollution exposure to noise (including road traffic, railways, commercial services and housing renovation) at the community level in Beijing. The non-uniform patterns of exposure to various types of noise pollution are evident. For instance, Figure 3A illustrates the proportion of residents reporting the road noise level in their surrounding environment as high or very high for each surveyed community. It suggests the cluster of communities with a high proportion (ranging from 45% to 70%) of high road traffic noise pollution is mainly located in the inner city and areas in the north, where car ownership has been higher than other areas and people make greater use of private vehicles [67]. Figure 3B–D present geographic variability of respondents’ pollution exposures to railway noise, commercial noise and housing renovation noise in Beijing, respectively. 

### 2.3. Measuring the Outcome Variables of Mental Health

**Figure 4:**
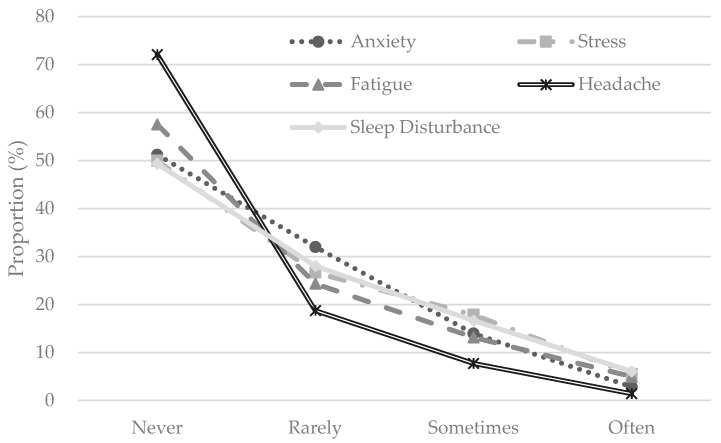
Population (%) in mental health categories.

In this study, we measured the self-reported symptoms of mental health or mental disorders with five dimensions: anxiety, stress, fatigue, headache, and sleep disturbance. These variables were measured by the frequency of being bothered by such feelings over the past four weeks through asking the questions: In general, how frequently have you suffered from the following mental health problems, such as anxiety, stress, fatigue, headache, and sleep disturbance, respectively. The responses are quantified on a 4-point scale: 1 = never, 2 = rarely, 3 = sometimes, and 4 = often. Figure 4 illustrates the proportion of the respondents in each category for different mental disorder variables. The majority (>70%) of respondents reported no feelings of headache in a recent month, while about half of the respondents reported they had suffered from the mental disorders of anxiety, stress and sleep disturbance. These results suggest that people more frequently suffer from the mental health problems of stress and sleep disturbance than headache in Beijing. To have better comparability with prior research in this area and facilitate the multilevel model implementation, the outcome variables of five mental health measures have been recoded into binary variables, respectively: 1 refers to having suffered from mental disorders (including rarely, sometimes and often) and 0 represents no such mental health symptoms (i.e., never).

### 2.4. Statistical Model

In our analysis, due to the two-level structure of our survey data (e.g., individual-level socio-demographics and community-level built environment characteristics) and the binary outcome variables of mental health, we employed a Bayesian multilevel logistic model to investigate the relationships between noise pollution and mental health. The five measures of mental health are modeled as binomial distributions with a logit link function, respectively. Let $p_{jk}$ represent the mental health status of individual *j* living in community *k*, and the Bayesian multilevel logistic model is expressed as [66,68]:

*Y_j_*_,*k*_ ~ Binomial (1, *p_jk_*); for *j* = 1, …, *J*; *k* = 1,…, *K*, (1)

$$\mathrm{Log}\;\{p_{jk}/(1-p_{jk})\}=\eta_{jk}=\alpha +P_{jk}^{\prime }\beta +S_{jk}^{\prime }\gamma +E_{jk}^{\prime }\delta +C_{k}^{\prime }\varphi +u_{k},\;u_{k}\;\sim \;N\;(0,\;\sigma^{2}),\{\alpha ,\;\beta ,\;\gamma ,\;\delta ,\;\varphi \}\;\sim \;N\;(0,\;b);\;\sigma^{2}\;\sim \;\mathrm{Inverse}\;\mathrm{Gamma}\;(e,f),$$

The log odds are related to a linear predictor ($\eta_{jk}$), which depends on a set of additive covariate effects. *P* represents perceived exposure to various types of noise pollution (road traffic, railways, commercial services and housing renovation), *S* refers to a set of socio-demographic attributes (e.g., age, gender, income, education, employment, marital status, residential status or migrants, housing tenure), *E* represents residents’ subjective evaluations of housing conditions (e.g., housing satisfaction) and community environment (traffic congestion and community attachment), and *C* includes the objective measures of the built environment at the community level (distances to the nearest subway station, main road, restaurant and park). 

Vectors of {α, ***β***, ***γ***, ***δ***, ***φ***} are fixed regression coefficients to be estimated, which quantify the effects of corresponding covariates on mental health on the logistic scale. Relatively diffuse priors are usually specified for fixed regression coefficients; for instance, a normal distribution with mean zero and very large variance (i.e., *b* = 10,000). The unobserved effect from community *k* on residents’ mental health is indicated by $u_{k}$, which follows a normal distribution with mean zero and variance $\sigma^{2}$ [69]. The prior distribution for the variance parameter $\sigma^{2}$ is an Inverse Gamma distribution with scale and shape parameters being *e* and *f*, following [69]. We note that given the above model specification, the regression coefficients are interpreted as cluster- or group-specific associations between independent variables and an outcome variable (i.e., the effect on the log-odds that Y equals to one from a one unit change in a predictor variable for a given community) [70,71].

The Bayesian multilevel logistic model was implemented using the R-INLA package (http://www.r-inla.org/), which is an interface of the C package INLA with R [72]. Five Bayesian multilevel logistic models were estimated for the five measures of self-reported mental health symptoms (anxiety, stress, fatigue, headache, and sleep disturbance), respectively. 

## 3. Results

### 3.1. Mental Disorders and Socio-Demographics

The estimates of odds ratios (OR) with corresponding 95% credible intervals (95% CI) from the five Bayesian multilevel logistic models are provided in Table 3. As shown in the table, most of the socio-demographic attributes are significantly associated with self-reported mental health symptoms or mental disorders in Beijing. Distinctness in odds of reporting various mental disorders is found between different age cohorts: middle-aged respondents (aged 40–49 years old) have significantly higher odds of reporting a wide range of mental health problems, including anxiety, stress, fatigue, headache, as well as sleep disturbance, than the young respondents (aged 18–30). Moreover, older respondents (aged 50 years old and above) tend to be more likely to suffer from sleep disturbance, fatigue and headache than the young respondents. 

A significant link between mental health and people with different levels of monthly income has been identified. Compared to respondents with medium-level income (between 6000 RMB and 10,000 RMB), the odds of reporting mental health problems for respondents with the highest income level increase by 45.6 %, 66.8% and 74.0% for anxiety, sleep disruption and fatigue, respectively, all else being equal. However, respondents with low income are not significantly distinguishable from medium-income respondents, which indicates a threshold effect of income on mental health. This suggests that the correlation between income and health is likely to be nonlinear. 

Gender, marital and migrant status are not significant covariates with various mental health symptoms, whereas employed residents are more likely to report the feelings of stress than their counterparts. Housing tenure is found to be significantly associated with some mental disorders, such as anxiety and sleep disturbance, while residents’ subjective evaluation of their housing conditions does not seem to make a significant difference to mental disorders, except for stress. The odds of reporting stress for residents with housing satisfaction significantly decrease by 30.1% compared to people who are not satisfied with their housing conditions. While many people cannot afford to purchase a commodity house with large floor space and good housing quality due to its very high price in Beijing, overall evaluation on housing satisfaction seems to be a significant predictor of stress or a main stressor for urban residents in Chinese megacities.

Moreover, with respect to the social environment, community attachment is found to be significantly associated with good mental health, as people with feelings of community attachment or social cohesion are less likely to report various mental health problems, particularly for anxiety and fatigue. This demonstrates that detachment from one’s community may be detrimental to mental well-being in Beijing.

### 3.2. Mental Disorders and Noise Pollution

Regarding the community-level objective built environment characteristics, proximity to the main road is a significant covariate of urban residents’ mental disorders in Beijing. Respondents residing in neighborhoods far from the main road have lower odds of reporting various mental health problems, such as anxiety, fatigue, and sleep disturbance, than residents living close to the main road. In contrast, accessibility to green space or parks is not significantly correlated with people’s mental health, although the correlations are positive. Other built environment variables, such as close proximity to public transit or commercial facilities like restaurants, which can be regarded as objective measures of exposure to railway noise or commercial noise, seem to be insignificant correlates of mental health in Beijing.

With respect to the subjective measures of perceived noise pollution, most of them are found to be significantly associated with self-reported mental health symptoms or mental disorders (Table 3). For instance, the odds of reporting fatigue for respondents with perceived high pollution exposure to road traffic noise increase by 62.7%, compared to people who rated the road noise pollution in their neighborhoods as very low. Residents who perceived higher exposure to railway or commercial noise tend to be more likely to suffer from mental disorders, such as anxiety, stress and sleep disturbance. These results demonstrate that perceived pollution exposure to multiple noise sources is negatively associated with self-reported mental health symptoms in Beijing, China.

Moreover, housing renovation noise pollution, which has rarely been investigated to date in the Chinese context, shows significant correlations with various types of mental health symptoms in Beijing. Respondents who perceived both moderate and high exposure to housing renovation noise tend to have significantly worse mental health, or more likely suffer from various mental disorders than those who reported very low housing renovation noise levels in their surrounding environment. Since the second-hand housing market is prosperous in urban Beijing, housing renovation is popular and widespread for urban residents. As a result, housing renovation noise has become a serious environmental problem in many Chinese cities, which has potential adverse effects on the population’s mental health.

### 3.3. Mental Disorders and Geographic Context

Geographic context effects, as measured by the distribution of community-level residuals, quantify how individuals’ mental health outcomes would vary across places net of their socio-demographic and economic characteristics. The geographic context effects on mental health are quantified by the median odds ratio (MOR), which transforms the community-level variance on the logit scale to a more interpretable odds ratio scale, thus offering an intuitive quantification of the magnitude of geographic context effects [73]. MOR is interpreted as the increased risks that would occur when moving residents from a low-risk area to a high-risk area. Here, it means an elevated odds of reporting mental health problems if an individual is relocated from a community with a small residual (i.e., low proportion of reporting mental disorders net of included covariate effects) to a community with a large residual. As shown in Table 3, the MOR values indicate that, on average, there is an increase of 52.0% (anxiety), 50.5% (stress), 42.0% (fatigue), 35.1% (headache), and 23.7% (sleep disturbance) in the odds of reporting mental health problems for the urban residents if they are relocated from a community (or a geographic context) that enhances mental health to one with undesirable environment. These results suggest a significant geographic context effect on mental well-being in Beijing, China.

## 4. Discussion

In prior studies, only the evidence on the associations between road traffic noise and sleep disturbance, annoyance, and cognitive performance was sufficient [13,15,18]. There has been little research concerning the linkage of noise pollution from housing renovation/construction and commercial services with a wide range of mental disorders, such as anxiety, stress, fatigue and headache. Even fewer studies have examined the spatial distribution of multiple noise pollution at fine geographic resolution and their associations with various mental health symptoms, especially in developing countries like China [5,9].

This study contributes to the literature in several dimensions. First, it presents the geographic variations in people’s subjective evaluations of noise pollution from diverse sources, including road traffic, railways, housing renovation, and commercial services at the community level in Beijing. It shows that road traffic noise and housing renovation noise are the principal noise stressors in the capital city of China, and there is geographic variability in pollution exposure to multiple noise sources. Second, this paper develops a broad conceptual framework to investigate the relationships between mental health and various objective and subjective measures, including socio-demographics, social environment, the objective physical built environment characteristics, and perceived noise-pollution evaluations [66,67]. It also examines the associations between these variables and multiple self-reported symptoms of mental health, such as anxiety, stress, fatigue, headache, as well as sleep disturbance. Finally, as mental health is associated with various factors at both the individual and community scales, Bayesian multilevel logistic models were employed to capture the hierarchical structure of our survey data and the unobserved effects of geographic context on the survey respondents [74].

Our results show that some social factors or socio-demographic attributes, such as age and income, are significant covariates of mental disorders for urban residents in Beijing. People with the highest income level have significantly increased odds of reporting mental health problems, such as anxiety, fatigue, and sleep disturbance. This seems to be in contrast with the findings from some prior studies, which found that people with high levels of income tend to have good health, although the correlation is likely to be nonlinear [66,75]. This result might be due to the potential bias of subjectively reported data on mental disorders and occupational stress for people with higher income levels [76]. Moreover, people with higher income levels have been found to suffer from more personal exposure to traffic-related air pollution, which might worsen their subjective health evaluations [77].

Housing tenure and satisfaction evaluation with housing conditions are significantly correlated with some mental health symptoms, such as anxiety, stress, as well as sleep disturbance. Social environment or community attachment is also a significant correlate of mental health, which is in agreement with previous findings [33]. Objective built environment variables seem to contribute little to the variation of self-reported mental disorders, except for proximity to the main road. People residing in neighborhoods close to the main road have significantly higher odds of reporting various mental health problems, such as anxiety, fatigue, and sleep disturbance.

Regarding the subjective measure of perceived noise exposure, it might produce a potential bias for different subpopulations. Therefore, before reporting the estimation results in Table 3, we ran a series of regression models to examine the associations between perceived noise pollution and some key socio-demographics, such as age, education and income, and found no systematic bias in our measurement of noise perception assessment. The Bayesian multilevel logistic modeling results show that residents’ perceived high exposures to road traffic noise tend to significantly increase their odds of reporting fatigue, while residents who perceived high exposure to railway noise are more likely to suffer from mental disorders such as anxiety, stress and sleep disturbance. Housing renovation and construction noise, which has rarely been investigated before, is a significant covariate of a wide range of mental health symptoms. As China is experiencing a rapid urbanization process, there are numerous ongoing construction projects that have led to an increase in environmental complaints, and construction noise has become a serious problem in many Chinese cities [5]. This type of noise pollution is a feature that reflects rapid urbanization in China and is different from the noise problems in Europe and North America, and thus needs more investigation in the Chinese context. Furthermore, the multilevel modeling results also suggest a significant geographic context effect on mental well-being in Beijing.

This study has some limitations. Since objective data on noise pollution at a fine geographic resolution is usually not available in China, it poses an important constraint on noise-health interaction research in the Chinese context. Although characteristics of the objective physical built environment may be regarded as proxies of people’s objective exposure to various noise sources, the inaccurate measure of objective noise exposure might weaken the mental health effects of the noise pollution in statistical models [47]. Moreover, due to the unavailability of some health-relevant confounders in the survey data, such as body mass index, smoking and drug use, these variables are missing in the analysis. However, the effects of such variables might be included in the residuals of the statistical models. Future research will need to collect both objective and subjective data on noise pollution at a fine spatiotemporal resolution to explore their combined effects on mental health, while controlling for a wide range of confounding variables.

## 5. Conclusions

China’s rapid urbanization and increase in car ownership have given rise to a wide range of environmental hazards, such as air and noise pollution, which pose significant health risks to the population. While the health effects of environmental pollution, particularly ambient air pollution, have received considerable attention in past research, the detrimental effects of noise pollution on people’s mental health need more investigation. This study attempts to shed light on the relationships between exposures to multiple sources of noise pollution and mental disorders in a Chinese megacity. Overall, road traffic and housing renovation are found to be the principal noise polluting sources influencing the urban living environment in Beijing. Higher noise-pollution exposures are significantly associated with the worse mental health of urban residents in general. The results also have some policy implications for the construction of transportation infrastructure, noise abatement interventions, and public health promotion. We argue that, while pursuing GDP growth and economic development in China, it is also important for the government to develop sustainable healthy environments and cities for promoting public health and well-being in the future.

## Figures and Tables

**Table 1:** Key socio-demographics and community environment evaluation in the survey.

Variables	Description	Proportion (%)
Gender	Female as the base category	50.0
Age	<30	11.5
	30–39	26.9
	40–49	18.5
	50–59	20.9
	60+	22.3
Monthly income (RMB)	<3000	7.9
	3000–6000	18.8
	6000–10,000	35.7
	10,000–15,000	14.8
	15,000+	22.7
Education	Primary	16.9
	Secondary	29.9
	Tertiary	53.2
Marital status	Married	84.6
Residence status	Migrants	28.2
Housing tenure	Owners	74.3
Employment	Employed	61.0
Housing satisfaction	Satisfied or very satisfied with housing	72.7
Community traffic congestion	Perceived serious or very serious traffic congestion around the community	74.7
Community attachment	Have feelings of community attachment	63.4

Note: RMB = renminbi, the official Chinese currency.

**Table 2:** Built environment characteristics of 26 surveyed communities.

Surveyed Communities	Distance to the Nearest Point of Interest (m)			
	Main Road	Park	Subway Station	Restaurant
Jin Yu Chi (JYC)	86.8	306.2	479.9	104.9
Xi Yuan Zi (XYZ)	120.8	378.5	462.6	77.3
Liang Jia Yuan (LJY)	167.4	1546.3	367.1	169.9
Xiang Lu Ying (XLY)	189.7	1623.0	325.7	152.7
Sheng Gu Zhuang (SGZ)	221.4	485.4	416.6	165.2
Ping Le Yuan (PLY)	374.2	86.7	708.4	93.1
Bai Zi Wan (BZW)	728.2	2185.3	936.1	7.5
Guan Dong Dian (GDD)	72.5	425.4	199.2	57.0
Gan Lu Yuan (GLY)	50.4	306.1	735.4	21.4
Jia Ming Yuan (JMY)	461.2	499.2	157.7	94.3
Mei He Yuan (MHY)	83.8	303.1	259.3	168.6
Zhu Fang (ZF)	257.4	665.8	819.1	18.0
Hua Yuan Lou (HYL)	441.9	769.3	1710.4	236.4
Xue Fu Shu (XFS)	63.7	605.2	951.2	146.4
Pu Hui Nan (PHN)	234.5	179.5	555.9	45.0
Pu Hui Si (PHS)	317.7	296.4	596.7	52.2
Jing Xi Bin Guan (JXBG)	86.9	614.7	499.9	98.2
Yun Yun Guo Ji (YYGJ)	348.1	770.9	1161.8	108.9
Xiao Yue Yuan (XYY)	796.8	1319.3	1237.8	297.4
Fei Cheng (FC)	220.0	502.1	1430.5	221.0
Sen Lin Da Di (SLDD)	151.0	1255.4	2320.3	267.4
Du Shi Fang Yuan (DSFY)	1918.4	1625.1	1847.8	416.6
Lv Zhou Jia Yuan (LZJY)	1885.5	771.9	1781.1	49.5
Hong Xing Lou (HXL)	69.5	853.7	2766.0	29.6
Qing Xin Yuan (QXY)	163.0	529.4	1749.7	58.9
Run Xing Jia Yuan (RXJY)	1216.6	297.2	1790.1	291.6

**Table 3:** Multilevel modeling results for five types of mental disorders.

Variables	Anxiety		Stress		Fatigue		Headache		Sleep Disturbance	
	OR	95% CI	OR	95% CI	OR	95% CI	OR	95% CI	OR	95% CI
Gender										
Female	1.000	reference	1.000	reference	1.000	reference	1.000	reference	1.000	reference
Male	0.919	0.706–1.196	0.993	0.761–1.295	0.840	0.648–1.090	0.888	0.667–1.181	0.839	0.649–1.084
Age										
<30	1.000	reference	1.000	reference	1.000	reference	1.000	reference	1.000	reference
30–39	1.208	0.715–2.045	1.028	0.605–1.739	1.433	0.848–2.443	1.303	0.704–2.471	**1.804**	**1.077–3.053**
40–49	**2.320**	**1.307–4.138**	**1.796**	**1.008–3.203**	**2.512**	**1.424–4.481**	**2.689**	**1.422–5.231**	**2.843**	**1.628–5.019**
50–59	1.376	0.745–2.546	1.069	0.577–1.980	**2.505**	**1.362–4.656**	**2.248**	**1.134–4.558**	**3.585**	**1.964–6.625**
60+	1.170	0.604–2.266	0.806	0.413–1.568	**2.759**	**1.427–5.394**	**3.266**	**1.573–6.951**	**3.127**	**1.631–6.061**
Income (RMB)										
<3000	1.069	0.632–1.803	1.350	0.795–2.293	0.837	0.492–1.410	1.557	0.908–2.646	1.221	0.734–2.040
3000–6000	0.836	0.573–1.217	0.875	0.598–1.276	0.912	0.626–1.322	1.391	0.935–2.064	0.949	0.660–1.360
6000–10,000	1.000	reference	1.000	reference	1.000	reference	1.000	reference	1.000	reference
10,000–15,000	**1.772**	**1.181–2.669**	**1.885**	**1.252–2.853**	1.469	0.986–2.188	1.092	0.686–1.716	1.351	0.912–2.004
15,000+	**1.456**	**1.011–2.101**	1.388	0.962–2.006	**1.740**	**1.214–2.499**	1.332	0.889–1.989	**1.668**	**1.171–2.382**
Education										
Primary	1.000	reference	1.000	reference	1.000	reference	1.000	reference	1.000	reference
Secondary	0.683	0.452–1.030	**0.506**	**0.331–0.768**	0.865	0.578–1.296	0.808	0.531–1.231	0.746	0.500–1.109
Tertiary	1.051	0.669–1.652	0.957	0.606–1.507	0.981	0.629–1.533	0.870	0.544–1.393	0.790	0.510–1.223
Employment status										
Unemployed	1.000	reference	1.000	reference	1.000	reference	1.000	reference	1.000	reference
Employed	1.003	0.671–1.497	**1.724**	**1.157–2.573**	1.279	0.862–1.907	1.053	0.687–1.620	1.028	0.696–1.521
Marital status										
Unmarried	1.000	reference	1.000	reference	1.000	reference	1.000	reference	1.000	reference
Married	0.778	0.499–1.208	0.788	0.503–1.231	0.818	0.528–1.266	0.915	0.569–1.486	0.715	0.463–1.098
Residence status										
Migrants	1.000	reference	1.000	reference	1.000	reference	1.000	reference	1.000	reference
Local residents	0.970	0.682–1.378	0.819	0.574–1.164	1.086	0.770–1.531	0.914	0.628–1.331	0.733	0.519–1.028
Housing tenure										
Renters	1.000	reference	1.000	reference	1.000	reference	1.000	reference	1.000	reference
Housing owners	**1.454**	**1.011–2.101**	1.294	0.898–1.873	1.215	0.853–1.740	1.249	0.848–1.867	**1.737**	**1.231–2.471**
Perceived community traffic congestion										
Not serious	1.000	reference	1.000	reference	1.000	reference	1.000	reference	1.000	reference
Serious	0.953	0.715–1.269	0.818	0.612–1.091	0.843	0.634–1.120	0.765	0.558–1.046	1.038	0.790–1.362
Housing satisfaction										
Unsatisfied	1.000	reference	1.000	reference	1.000	reference	1.000	reference	1.000	reference
Satisfied	0.947	0.687–1.308	**0.699**	**0.505–0.966**	0.872	0.640–1.190	0.778	0.563–1.080	0.937	0.693–1.270
Community attachment										
No such feelings	1.000	reference	1.000	reference	1.000	reference	1.000	reference	1.000	reference
Have such feelings	**0.736**	**0.549–0.984**	0.913	0.681–1.224	**0.677**	**0.507–0.901**	0.872	0.638–1.193	0.878	0.662–1.164
Standardized distance to the main road	**0.780**	**0.605–0.995**	0.789	0.614–1.003	**0.796**	**0.634–0.989**	0.828	0.666–1.024	**0.791**	**0.660–0.942**
Standardized distance to the nearest park	1.091	0.864–1.382	1.117	0.887–1.411	1.001	0.812–1.236	1.021	0.835–1.254	1.049	0.889–1.238
Standardized distance to the nearest subway station	1.123	0.881–1.436	1.044	0.821–1.327	1.157	0.932–1.441	1.069	0.866–1.317	1.005	0.846–1.193
Standardized distance to the nearest restaurant	0.897	0.710–1.126	1.010	0.805–1.266	0.932	0.758–1.145	1.001	0.820–1.226	1.012	0.859–1.192
Perceived noise pollution										
Very low road noise	1.000	reference	1.000	reference	1.000	reference	1.000	reference	1.000	reference
Moderate road noise	0.899	0.617–1.311	0.886	0.605–1.296	1.378	0.943–2.023	1.165	0.760–1.801	0.970	0.674–1.394
High road noise	0.667	0.435–1.021	1.045	0.683–1.599	**1.627**	**1.067–2.492**	1.534	0.962–2.467	0.771	0.513–1.157
Very low train noise	1.000	reference	1.000	reference	1.000	reference	1.000	reference	1.000	reference
Moderate train noise	1.257	0.888–1.781	1.193	0.839–1.699	1.100	0.779–1.554	1.016	0.694–1.482	1.034	0.737–1.452
High train noise	**2.659**	**1.639–4.354**	**2.272**	**1.390–3.758**	1.404	0.886–2.230	1.447	0.894–2.347	**1.854**	**1.178–2.955**
Very low commercial noise	1.000	reference	1.000	reference	1.000	reference	1.000	reference	1.000	reference
Moderate commercial noise	**1.793**	**1.296–2.485**	**1.549**	**1.116–2.151**	1.307	0.947–1.805	**1.513**	**1.060–2.164**	**1.558**	**1.139–2.133**
High commercial noise	0.951	0.560–1.607	0.943	0.548–1.614	1.018	0.610–1.693	1.555	0.905–2.655	1.330	0.797–2.214
Very low renovation noise	1.000	reference	1.000	reference	1.000	reference	1.000	reference	1.000	reference
Moderate renovation noise	**2.572**	**1.805–3.689**	**2.053**	**1.444–2.931**	**1.792**	**1.264–2.552**	1.301	0.884–1.924	**1.632**	**1.167–2.289**
High renovation noise	**2.625**	**1.756–3.951**	**2.263**	**1.517–3.394**	**2.057**	**1.390–3.058**	1.401	0.908–2.166	**1.494**	**1.023–2.184**
Community-level variance	0.194		0.185		0.136		0.100		0.050	
Median Odds Ratio (MOR)	52.0%		50.5%		42.0%		35.1%		23.7%	

Note: Bold font reflects statistically significant results at *p* < 0.05. OR represents odds ratios (median) and 95% CI refers to the 95% credible interval in the Bayesian inference paradigm.

## Statement

There is no human subject protection or ethics requirements in China concerning this research, so a project identification code is not available. The data were collected from the participants with their consent.
